# Imaging Brain Function with Functional Near-Infrared Spectroscopy in Unconstrained Environments

**DOI:** 10.3389/fnhum.2017.00258

**Published:** 2017-05-17

**Authors:** Joana B. Balardin, Guilherme A. Zimeo Morais, Rogério A. Furucho, Lucas Trambaiolli, Patricia Vanzella, Claudinei Biazoli, João R. Sato

**Affiliations:** ^1^Center of Mathematics Computation and Cognition, Universidade Federal do ABCSão Bernardo do Campo, Brazil; ^2^Instituto do Cérebro, Hospital Israelita Albert EinsteinSão Paulo, Brazil; ^3^NIRx Medizintechnik GmbHBerlin, Germany

**Keywords:** fNIRS, brain imaging, naturalistic experimentation, wearable, sports, musicians, hyperscanning, continuous monitoring

## Abstract

Assessing the neural correlates of motor and cognitive processes under naturalistic experimentation is challenging due to the movement constraints of traditional brain imaging technologies. The recent advent of portable technologies that are less sensitive to motion artifacts such as Functional Near Infrared Spectroscopy (fNIRS) have been made possible the study of brain function in freely-moving participants. In this paper, we describe a series of proof-of-concept experiments examining the potential of fNIRS in assessing the neural correlates of cognitive and motor processes in unconstrained environments. We show illustrative applications for practicing a sport (i.e., table tennis), playing a musical instrument (i.e., piano and violin) alone or in duo and performing daily activities for many hours (i.e., continuous monitoring). Our results expand upon previous research on the feasibility and robustness of fNIRS to monitor brain hemodynamic changes in different real life settings. We believe that these preliminary results showing the flexibility and robustness of fNIRS measurements may contribute by inspiring future work in the field of applied neuroscience.

## Introduction

Despite the immense success of functional Magnetic Resonance Imaging (fMRI), advancements in the field of neuroimaging are founded on the complementary strengths of acquisition techniques that access different aspects of neural, metabolic and hemodynamic activities (Poldrack and Farah, 2015). Functional near-infrared spectroscopy (fNIRS) has been providing increasingly reliable and robust measures of localized hemodynamic responses in cortical surface (Boas et al., 2014; Strait and Scheutz, 2014). Based on the differential optical properties of hemoglobin states in the near-infrared spectrum, fNIRS can infer changes on local concentration of oxy and deoxy-hemoglobin. These measures are fairly comparable to BOLD (blood oxygenation level dependent) signal and have the additional advantage of representing more directly interpretable physiological signals. In addition, the neurovascular coupling phenomenon allows for interpreting changes in oxy and deoxyhemoglobin concentrations in terms of neural activity. Several studies have compared fNIRS measures with the BOLD based fMRI in different experimental settings, showing notable superposed hemodynamic signals and convergent functional mappings (Cui et al., 2011), even in more naturalistic situations (Noah et al., 2015).

The fNIRS has attracted attention especially due to the possibility of overcoming some limitations associated with the MRI environment and muscular/movement artifacts in Electroencephalography (EEG). For instance, the portability and relative low-cost of fNRIS equipment might be essential to extend neuroimaging studies to underprivileged or isolated communities (Lloyd-Fox et al., 2014). Particularly, the advance of multi-channel and portable fNIRS hardware has enabled neuroimaging studies in more naturalistic settings. Examples of studies applying experimental procedures and paradigms unsuitable in the MRI environment include: reaching for objects or stepping in place (Nishiyori et al., 2016), setting and clearing a table (Koehler et al., 2012), engaging in face to face conversation (Suda et al., 2011; Jiang et al., 2012; Takei et al., 2013, 2014; Lloyd-Fox et al., 2015), giving a speech (Tuscan et al., 2013), watching live and televised actions (Shimada and Hiraki, 2006), using tools (Helmich et al., 2015), performing inter-individual actions coordinately (Egetemeir et al., 2011), accessing prospective memory while walking (Pinti et al., 2015) and actually driving on a highway (Yoshino et al., 2013). fNIRS has also been proposed as an alternative to uncovering cognitive and affective states for real-life application of brain-computer interface (reviewed in Strait and Scheutz, 2014).

Despite the fact that several studies have used fNIRS to perform more naturalistic experiments out of lab, we believe that possible applications of fNIRS in real-life situations are still underestimated. At least three characteristics of fNIRS-based naturalistic experimentation might contribute to foster cognitive neuroscience: (i) the possibility of studying aspects of cognition that remains underappreciated in restricted environments; (ii) a way to increase generality of results by replicating neuroimaging experiments in a variety of settings; (iii) and the expansion of potential applications of findings obtained with fMRI. Moreover, naturalistic experiments are possible due to the relative robustness of fNIRS signal against head motion artifacts when compared with EEG and fMRI. Considering these features, we argue in favor of fNIRS studies in real-life situations and here we show illustrative applications for practicing a sport, playing a musical instrument alone or in duo and performing daily activities for several hours. With these applications we aim to illustrate that fNIRS is even more feasible in naturalistic experimentation than what is usually proposed. We also discuss the remaining pitfalls and limitations that need to be addressed in fNIRS-based naturalistic experiments.

## Principles and instrumentation

### fNIRS and local activity

The assessment of brain activity through the fNIRS technique relies on the neurovascular-coupling phenomenon, similarly to fMRI. The technique relies on the relationship between neural activity and changes in regional cerebral blood flow (rCBF) affecting oxygenation and hemoglobin content in blood vessels. The physiological and physical principles of the technique have been extensively described elsewhere (Obrig and Villringer, 2003; Scholkmann et al., 2014). Both fMRI and fNIRS are grounded on the model that states that an increase in oxygen metabolism due to neural activity is accompanied by a disproportional increase in rCBF causing a local hyperoxygenation (Fox and Raichle, 1986). This cascade of events is reflected in an increase in the concentration of oxy-hemoglobin and a decrease in concentration of deoxyhemoglobin (Chance and Villringer, 1997). fNIRS takes advantage to the fact that these hemoglobin chromophores are selective absorbers of light in the near infrared part of the spectrum, while biological intervening tissues such as bone and skin are transparent. Therefore, the principle of the technique is to non-invasively quantify the light migrating from sources to detectors positioned in the scalp taking into account these different absorption and scattering properties.

### Hardware and data acquisition

The hemodynamic changes for the present study were obtained from the optical changes collected using two different continuous-wave, functional near-infrared spectroscopy systems (NIRScout16x16 and NIRSport8x8, NIRx Medical Technologies, Glen Head, NY). Only one system has been used per experiment and its choice was primarily based on: (i) number of subjects per run (i.e., single subject or hyperscanning); (ii) number and size of cortical area(s) of interest; and (iii) whether movement of the entire body was expected during the measurement.

The NIRScout system, consisting of 16 LED illumination sources (760 and 850 nm) and 16 fiber optic detectors, was used to collect data in the experiment based on hyperscanning (violin duo) and to achieve coverage of frontal and motor cortical areas (piano playing). The NIRSport wearable system with 8 LED sources (760 and 850 nm) and 8 active detectors was used in the table-tennis and in the continuous monitoring in daily activities experiments. The sampling rate for the experiments was 7.81 Hz, with the exception of the piano measurement, which was 3.91 Hz.

**Figure 1:**
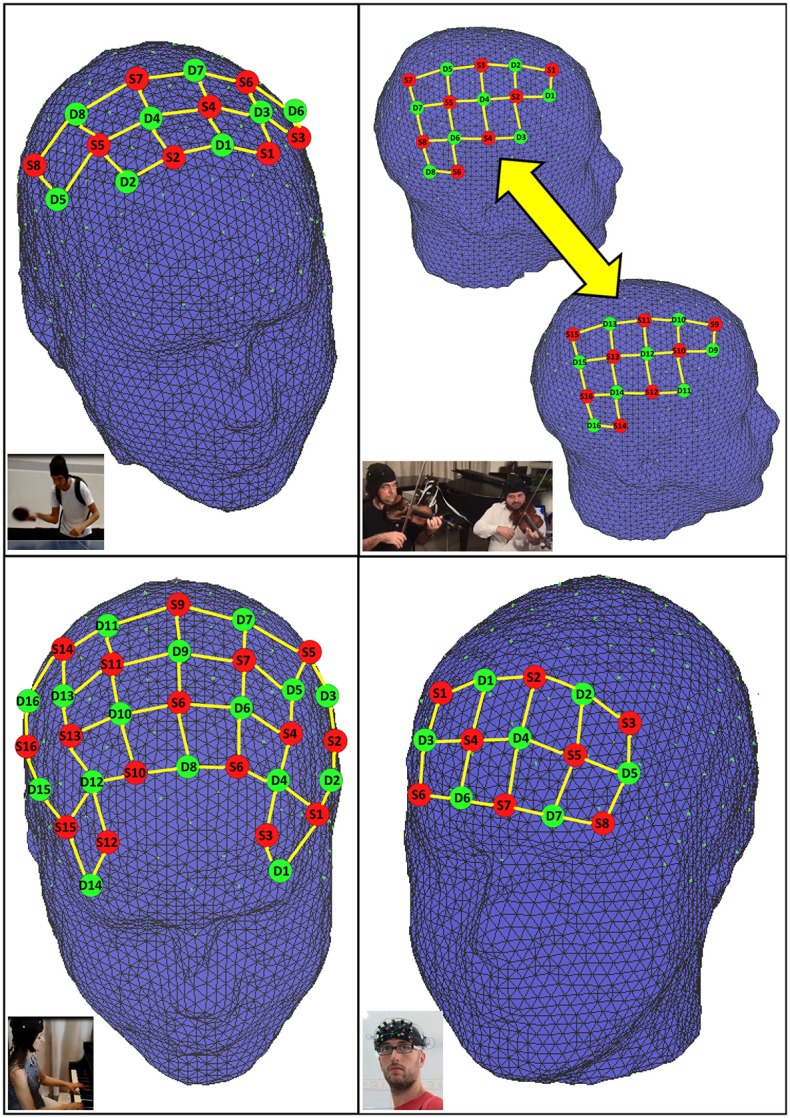
**fNIRS probe setup used in each experiment. Top left:** supplementary motor and primary motor cortex (23 channels). **Top right:** hyperscanning right hemisphere motor and temporo-parietal junction (22 channels/subject). **Bottom left**: dorsolateral prefrontal and primary motor cortex (51 channels). **Bottom right**: forehead headband covering prefrontal cortex (22 channels).

Sources and detectors were placed on the region(s) of interest of the measuring cap (10-5 international system, except for the continuous measurement, which used a frontal headband). Their spatial distribution on the cap was chosen to result in channels (i.e., source-detector pairs) with standard inter-optode distances of approximately 30 mm, thus guaranteeing an optimal balance between signal-to-noise ratio and sensitivity to the cortex (Li et al., 2011; Strangman et al., 2013). The setups used in each experiment and the corresponding number of channels are depicted in Figure 1.

## Illustrative experiments

In this perspective article, we describe four illustrative experiments emphasizing the potential of fNIRS in naturalistic experimentation. These are proof-of-concept experiments in different case-studies: (i) playing sports (table tennis); (ii) playing the piano; (iii) human-interaction (violin duo with hyperscanning) and (iv) continuous monitoring in daily activities. Previous studies have already described fNIRS applications to study real life situations, although with different protocols and aims. For example, Piper et al. (2014) demonstrated the use of functional NIRS brain imaging during outdoor bicycle riding. The fNIRS potential to investigate brain-to-brain coupling during natural verbal communication between storytellers and listeners have also been recently reported (Liu et al., 2017). Finally, Galderisi et al. (2016) reported on the use of this brain imaging technique for a long-term 7-day continuous monitoring of the neonatal brain.

The description and results of each one of our proposed new experiments are presented in the following subsections. The probes layout and hardware used in each experiment were described in the previous section. In all experiments, the signal of each channel was temporally filtered with a band-pass filter (0.01–0.2 Hz), to remove cardiac and respiratory frequencies as well as very low frequency artifacts. The oxy-Hb and deoxy-Hb concentration changes were calculated by using the modified Beer-Lambert equation (Delpy et al., 1988). All these preprocessing steps and the statistical analysis described in subsequent sections were carried out using the software nirsLAB_v201412 (Xu et al., 2014). In the current illustrations, we only show the results for oxyhemoglobin signals for the sake of brevity and since concentration changes of this molecule are very sensitive to changes in rCBF (Hoshi et al., 2001).

### Table tennis

In this first illustrative experiment, we show the feasibility of fNIRS recording during physical activities that require moderate levels of motion (Supplementary Material, video Table Tennis). This was a table tennis trial in which the participant was an athlete, male, 30 years old, right-handed and with 16 years of regular practice. The experiment consisted of a block design with three active conditions: forehand, predictable and unpredictable conditions alternated with rest (baseline). The order of active conditions was randomized and each block had the duration of 20 s (30 s for rest) and was repeated 10 times. In the forehand condition, the ball was always directed to the right side of the table of the participant, while in the predictable condition the ball was directed twice to the right side and then twice to the left side (thus inducing a backhand hit). In the unpredictable condition, there were no prior fixed locations (right or left) for the hit. All the hits were returned to the right side of the partner (J.R.S) at the other end of the table. Here we aimed to identify cortical regions which are more active in unpredictable than in predictable condition. In order to do so, the oxy-Hb and deoxy-Hb time courses of each channel were modeled with a general linear model (GLM), with a correction for serial autocorrelations using the hemodynamic response function (HRF) precoloring method (Friston et al., 2000) on the downsampled signal at 1 Hz. We computed the contrast of the HRF betas estimates between unpredictable and predictable conditions, and results for each channel were Bonferroni corrected for multiple comparisons.

In addition, we also evaluated the coefficient-of-variation as a metric for the signal quality across the four conditions. The CV (CV = standard deviation / mean) of each wavelength was calculated at each frame considering a sliding-window of 5 s width (yielding 21 frames, centered at the frame of interest). This is a similar procedure as used and described by Piper et al. (2014) to evaluate the influence of motion artifacts during physical exercise. The motor/premotor setup was used in this experiment (Figure 1, top-left). Our hypothesis was that left motor/premotor regions would be more active in the unpredictable condition.

**Figure 2:**
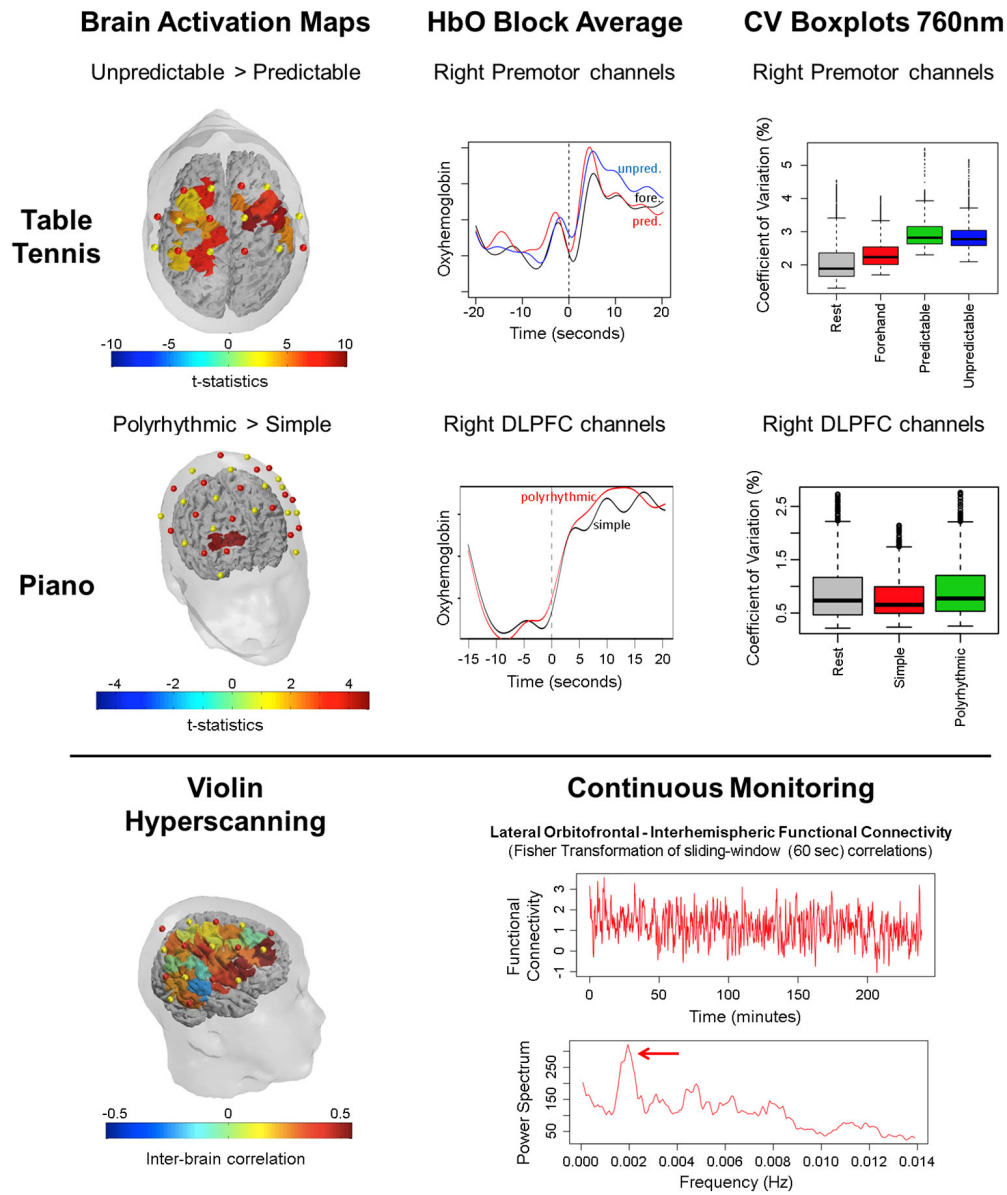
**Results of the four illustrative experiments. Top:** Table tennis experiment - cortical activation maps based on oxyhemoglobin, average signal from rest to task (zero is the start of the task) and CV boxplots for each condition. **Middle**: Piano-playing experiment. **Bottom**: violin duo experiment - intersubjects correlation maps (left); and Continuous monitoring experiment (right) – time-varying interhemispheric correlations between lateral orbitofrontal cortex signals and the corresponding estimated power spectrum.

The results are shown in Figure 2 (top) (see Table S1, Figure S1 in the Supplementary Material for statistical results of all channels). As expected, the statistical parametric maps highlight that these regions were indeed more active during the unpredictable when compared to the predictable condition. Interestingly, the right premotor cortex was also more active, suggesting that location uncertainty required motor preparation in both hemispheres. Moreover, the boxplots suggest that the signal reliability was similar between the two conditions. However, as expected, the coefficient-of-variation was slightly smaller during rest (~1% less).

### Playing the piano

In this second illustrative experiment, we highlight the feasibility of fNIRS recording during the performance of two musical excerpts in the piano and we compared cortical activation levels in rhythmically simple vs. polyrhythmic conditions (Supplementary Material, video Piano). Both excerpts were 30 s long and corresponded to the first bars of the following compositions by Alexander Scriabin: Etude op. 42, no. 5, in C sharp minor, and Etude op. 8, no. 2, in F sharp minor, for the rhythmically simple vs. polyrhythmic conditions, respectively. We used a block-design with two active conditions (simple vs. poly) alternated with rest (baseline). The order of conditions was randomized. Each cycle had the duration of 20 s and was repeated 10 times. The participant (P.V.) was a professional pianist, 51 years old, female, right-handed with 45 years of regular practice. Statistical parametric maps of activation and the CV metric were computed in a similar fashion as the Table Tennis experiment described above. The setup is depicted in Figure 1 (bottom-left).

Figure 2 (middle) depicts the results suggesting that the right dorsolateral prefrontal cortex (DLPFC) was more active in the polyrhythmic than in the rhythmically simple condition (see Table S2, Figure S2 in the Supplementary Material for statistical results of all channels). This finding points toward a greater cognitive effort and sustained attention in polyrhythmic playing. The coefficients-of-variation shown in the boxplots suggest a satisfactory signal quality during the acquisition of the data.

### Human interaction during a violin duo

In this third case study, we investigated the temporal patterns of cortical activation during human interaction when playing a violin duo with simultaneous fNIRS acquisition from both musicians (each of them playing a different musical line) (Supplementary Material, video Violin Duo). The participants (professional level, 41 and 50 years old, males, right-handed with 28 and 40 years of regular practice, respectively) were instructed to play a 30 s excerpt of Antonio Vivaldi's Allegro from the Concerto No 1 in E Major, Op. 8, RV 269, "Spring". We used the setup described in Figure 1 (top-right).

We analyzed the cortical activation synchronization between subjects. Thus, for each channel of the two violinists, we carried out a correlation analysis of the oxyhemoglobin concentration during the performance of this piece. An interbrain correlation map was built based on the Spearman correlation coefficient, as shown in Figure 2 (bottom-left; see Table S3, Figure S3 in the Supplementary Material for statistical results of all channels). Interestingly, higher synchronization between the brains of the two violinists occurred in parietal and frontal regions, in premotor and somatomotor areas.

### Continuous monitoring

Finally, we demonstrate the possibility of using fNIRS for long periods of data acquisition. In this concept proof, the participant (G.Z.M.), a 26 years old male (right handed) was monitored during approximately 4 h, from 10 a.m. to 2 p.m., while carrying out daily activities related to his work (mostly software coding and writing technical support emails). We used a prefrontal setup, as described in Figure 1 (bottom-right).

A 1-min sliding window was used to quantify the interhemispheric functional connectivity of the lateral orbitofrontal cortex. The Spearman correlation coefficient between the oxyhemoglobin concentration signals was obtained for each window, providing a time series for the interhemispheric functional connectivity. This time-varying connectivity and its respective estimated power spectrum (smoothed periodogram) are shown in Figure 2 (bottom-right). Interestingly, the spectrum suggests a periodicity with peaking at 0.002 Hz. We prefer to avoid conjecturing on the physiological interpretation of this finding, but this specific frequency seems to not be directly related to a non-functional component (e.g., physiological noise). However, it is important to mention that a previous resting state fMRI study has already reported cyclic fluctuations in functional connectivity (Handwerker et al., 2012).

## Limitations and future directions

We described a series of proof-of-concept experiments examining the potential of fNIRS in assessing the neural correlates of cognitive and motor processes in unconstrained environments. Our results build on previous research assessing the feasibility and robustness of fNIRS to monitor brain hemodynamic changes in real work settings (Ayaz et al., 2013; Pinti et al., 2015). Therefore, though preliminary, our results highlight that fNIRS is a promising tool for recording brain functional measures in experiments with more ecological validity. Although local oxygen concentration is an indirect measure of metabolic activity, fNIRS is very attractive due to the robustness against muscular, head motion artifacts, external electrical noise, and the possibility of data acquisition free from magnetic environment. In this sense, we believe that fNIRS should not be considered a competitor of EEG or fMRI, but an alternative modality for cases in which these techniques are not suitable or cannot be used.

However, important limitations should be mentioned. First, not all brain regions can be reached with fNIRS. Generally, only cortical regions beneath the scalp can be reliably and directly measured, thus excluding subcortical and other deep cortical structures (Patil et al., 2011). Second, though the temporal sampling rate might be relatively high, the changes in oxygen levels depend not only on the metabolic activity but also on a slow hemodynamic coupling process. Consequently, the traditional experimental designs using fNIRS might be more similar to the ones used in fMRI studies. Third, the interference of superficial veins/arterioles and extracortical components are challenging obstacles in terms of signal specificity (Brigadoi and Cooper, 2015; Tachtsidis and Scholkmann, 2016). Finally, though the method is robust to head motion, caution must be taken in experiments that may induce venous pooling or orthorstatic hypotension (e.g., squatting or lying down and then standing up).

Also, the technological and methodological advances in fNIRS are fast and still in progress. Three-dimensional functional brain mapping via fNIRS based on diffuse optical tomography is a recent and growing field (Bluestone et al., 2001). Advancements in hardware, such as the improvements in avalanche photodiodes (Scholkmann et al., 2014), are additional promising developments regarding signal quality. The scalability and reduction in the hardware size might also be relevant to make fNIRS devices not only portable but also wireless, allowing for an even more naturalistic data acquisition. Finally, further research to quantify the impact of other factors and components on fNIRS signals as expected in open environment scenarios, e.g., blood pressure (Kirilina et al., 2012), respiratory changes (Scholkmann et al., 2013) and gravitational pulling (Smith et al., 1985), seems to be important to achieve a solid understanding of the obtained results. This knowledge would be essential to enable reliable fNIRS experiments in extreme unconstrained applications, e.g., bungee jumping or skydiving.

To conclude, we believe that the flexibility and robustness of fNIRS measurements make it a promising tool for naturalistic experimentation. This is an attractive feature when considering the potential applications of neuroscience in the fields of educational and sports sciences, as well as in clinical studies in neurology and biological psychiatry.

## Ethics statement

All participants gave informed written consent in accordance with ethics approval by the Universidade Federal do ABC Ethics Committee, Bernardo do Campo, Brazil. The participants provided written consent to appear in the image publish.

## Author contributions

Study concept and design: JB, PV, and JS. Acquisition, analysis, or interpretation of data: JB, GZ, RF, PV, CB, and JS. Statistical analysis: JS. Drafting of the manuscript: JB, GZ, PV, CB and JS. Administrative, technical, or material support: GZ, RF, and LT.

## Funding

LT was supported by State of São Paulo Research Foundation—FAPESP Fellowship (2015/17406-5).

### Conflict of interest statement

GZ is employed by NIRx Medizintechnik GmbH. The other authors declare that the research was conducted in the absence of any commercial or financial relationships that could be construed as a potential conflict of interest.
