# Supplementary material for: Imaging Brain Function with Functional Near-Infrared Spectroscopy in Unconstrained Environments
Source: Front Hum Neurosci. 2017 May 17;11:258. doi: 10.3389/fnhum.2017.00258 (PMC5434677; doi:10.3389/fnhum.2017.00258)
Supplement: Supplementary file 4 [file DataSheet1.docx]

Table S1. T-values of each channel for the contrast between the unpredictable and the predictable experimental condition. Significant signal changes (Bonferroni corrected p<0.0021) for oxyhemoglobin concentrations are highlighted in bold.

| Channel # | t-value |
| --- | --- |
| **1** | **7.43** |
| 2 | 3.17 |
| 3 | 2.75 |
| 4 | 0.44 |
| **5** | **5.14** |
| **6** | **4.76** |
| 7 | 0.32 |
| 8 | -0.06 |
| **9** | **4.63** |
| 10 | 1.25 |
| **11** | **7.26** |
| **12** | **7.10** |
| **13** | **10.05** |
| **14** | **9.31** |
| 15 | 0.72 |
| 16 | 2.25 |
| 17 | 3.58 |
| 18 | 7.49 |
| 19 | -0.65 |
| 20 | -0.56 |
| 21 | 2.16 |
| **22** | **5.00** |
| 23 | 2.70 |


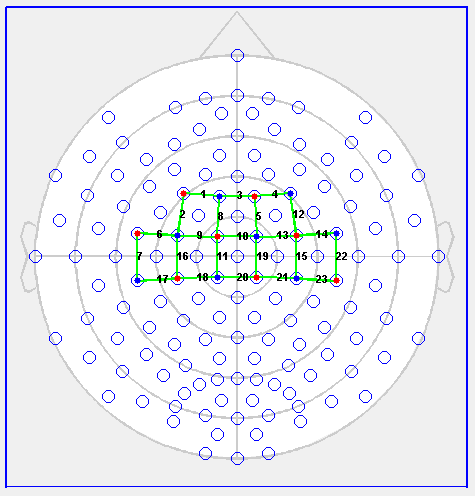


Figure S1. Channels’ numbers of the montage used for the table tennis experiment.

Table S2. T-values of each channel for the contrast between the polyrythmic and the simple experimental condition. Significant signal changes (Bonferroni corrected p<0.0009) for oxyhemoglobin concentrations are highlighted in bold.

| Channel # | t-value |  | Channel # | t-value |
| --- | --- | --- | --- | --- |
| 1 | 1.71 |  | 27 | 0.84 |
| 2 | 1.61 |  | 28 | -0.05 |
| 3 | 1.13 |  | 29 | 0.67 |
| 4 | -3.29 |  | 30 | 2.97 |
| 5 | -1.56 |  | 31 | -3.15 |
| 6 | -0.10 |  | 32 | 1.83 |
| 7 | 2.72 |  | 33 | -2.16 |
| 8 | 1.38 |  | 34 | -0.79 |
| 9 | 0.29 |  | 35 | -0.39 |
| 10 | -0.23 |  | 36 | 0.67 |
| 11 | -1.50 |  | **37** | **4.65** |
| 12 | -0.77 |  | 38 | 2.00 |
| 13 | -0.38 |  | 39 | -0.08 |
| 14 | -0.55 |  | 40 | 1.97 |
| 15 | 0.26 |  | 41 | -0.32 |
| 16 | 0.90 |  | 42 | -0.63 |
| 17 | 0.58 |  | 43 | -0.09 |
| 18 | 0.59 |  | 44 | 0.39 |
| 19 | -1.36 |  | 45 | 1.13 |
| 20 | -0.14 |  | **46** | **4.68** |
| 21 | -0.30 |  | 47 | 2.67 |
| 22 | -2.49 |  | 48 | 1.97 |
| 23 | 0.24 |  | 49 | 0.45 |
| 24 | 1.15 |  | 50 | -6.39 |
| 25 | -1.99 |  | 51 | 2.11 |
| 26 | -2.85 |  |  |  |
|  |  |  |  |  |
|  |  |  |  |  |


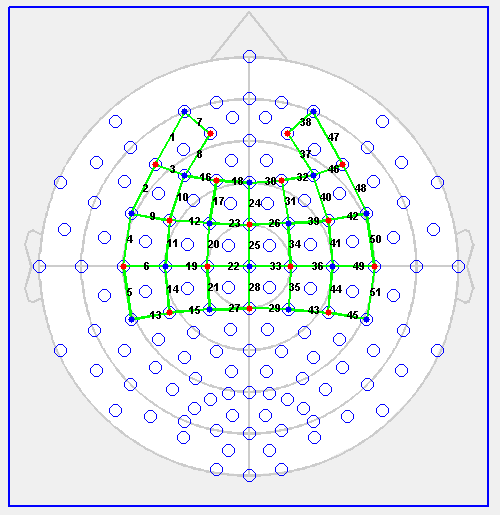


Figure S2. Channels’ numbers of the montage used for the playing the piano experiment.

Table S3. Mean Spearman rank correlation of the oxyhemoglobin concentration between each channel of the violinists during the performance of the 30 seconds Vivaldi excerpt. Higher correlation values (highlighted in bold) suggest higher interbrain synchronization in channels located over the parietal and frontal regions, as well as in premotor and somatomotor areas.

| Channel # | Spearman rho |
| --- | --- |
| **1** | **0.55** |
| 2 | -0.03 |
| **3** | **0.31** |
| 4 | 0.04 |
| **5** | **0.52** |
| 6 | 0.29 |
| 7 | 0.29 |
| 8 | 0.09 |
| 9 | 0.16 |
| **10** | **0.39** |
| **11** | **0.32** |
| 12 | -0.10 |
| **13** | **0.40** |
| **14** | **0.32** |
| 15 | -0.05 |
| 16 | 0.25 |
| 17 | -0.25 |
| **18** | **0.30** |
| 19 | 0.08 |
| 20 | 0.31 |
| 21 | 0.28 |
| 22 | 0.00 |
| 23 | 0.24 |


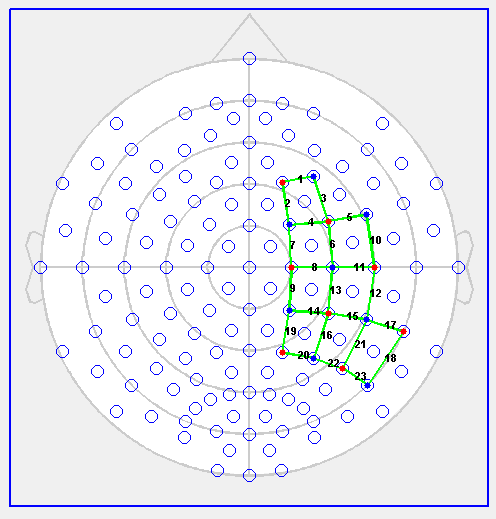


Figure S3. Channels’ numbers of the montage used for the hyperscanning experiment.
